# Fludarabine-resistance associates with ceramide metabolism and leukemia stem cell development in chronic lymphocytic leukemia

**DOI:** 10.18632/oncotarget.26043

**Published:** 2018-09-04

**Authors:** Chunfa Huang, Yifan Tu, Carl E. Freter

**Affiliations:** ^1^ Division of Hematology/Oncology, Department of Internal Medicine, School of Medicine, Saint Louis University, Saint Louis, MO, 63110, USA

**Keywords:** ceramide, chronic lymphocytic leukemia, drug-resistance, fludarabine, glucosylceramide

## Abstract

Fludarabine (flu) -containing regimens such as flu, cyclophosphamide and rituximab have been established as one of the standard first line therapy in medically-fit chronic lymphocytic leukemia (CLL) patients. Therefore, flu-refractory (primary flu-insensitivity or flu-caused relapse) remains a major problem causing treatment failure for CLL patients. We isolated the peripheral blood mononuclear cells (PBMCs) from CLL patients and treated with flu to find flu-refractory cases, and established flu-resistant clonal cells to study molecular mechanism of flu-resistance. By comparing parental MEC-2 cells, a human CLL cell line, we found that flu-resistant clonal cells were significantly increased lethal dose 50 of flu concentration, and up-regulated expression of P-glycoprotein, a drug-resistant marker, glucosylceramide synthase (GCS), an enzyme that can convert ceramide to glucosylceramide, and CD34, a leukemia stem cell marker. Overexpression of GCS leads to promptly elimination of cellular ceramide levels and accumulation of glucosylceramide, which reduces apoptosis and promotes survival and proliferation of flu-resistant clonal cells. Furthermore, we demonstrated that the accumulation of glucosylceramide can be blocked by PDMP to restore flu-sensitivity in flu-resistant clonal cells. We also found that elevating glucosylceramide levels in flu-resistant clonal cells was associated with up-regulation of GCS and CD34 expression. Importantly, overexpression of GCS or CD34 was also determined in flu-refractory PBMCs. Our results show that flu-resistance is associated with the alteration of ceramide metabolism and the development of leukemia stem cell-like cells. The flu-resistance can be reversed by GCS inhibition as a novel strategy for overcoming drug resistance.

## INTRODUCTION

Chronic lymphocytic leukemia (CLL) is classified as a lymphoproliferative disorder characterized by the accumulation of a clonally expanded lymphocytic population with resistance to apoptosis and co-expression of CD5, CD19, and CD23 in B lymphocytes in the peripheral blood, lymph nodes, bone marrow, spleen and liver [1–4]. Cytogenetic analysis indicates that CLL has many different genetic mutations which are heterogeneous in terms of progression, therapeutic response and outcome [5–8]. Several biological markers related to CLL outcome have been identified such as deletion of chromosome 17p13, 11q23 and 13q14, trisomy 12, expression of ZAP70, IgVH genomic rearrangement, and aberration of tumor protein 53 gene [5–8]. These cytogenetic markers allow the stratification of broad prognostic groups of CLL patients; however, underlined mechanisms of drug insensitivity (primary drug refractory and chemo-caused drug-relapse) and the regulation to overcome drug-resistance remain poorly understood.

Since 1995, fludarabine (flu) has been used as one of the chemotherapy agents to treat CLL [9]. Later, rituximab, a monoclonal antibody against B-cell marker CD20, and alemtuzumab, a monoclonal antibody that binds to CD52, were also developed and have been used for immunotherapy in chemo naïve patients [10, 11]. More recently, in order to reduce the relapse rate and increase complete responses, a combination of multiple therapeutic agents such as the combination of flu, cyclophosphamide and rituximab (FCR) has been developed [12–14]. FCR has been established as one of the current standard first line treatment for medically-fit CLL patients. The medium survival of FCR treatment is longer than 10 years, however, the survival for flu-refractory patients is only 0–3 years [10, 12]. Furthermore, most of the treated patients will eventually relapse, and about 10% of CLL patients are primary flu-refractory [10, 12]. It is clear that flu-insensitivity (primary flu-refractory and flu-caused relapse) is associated with poor survival, and represents a big challenge for treatment used flu and other purine analogue drug containing regimens. Therefore, it is very important to understand molecular mechanisms of flu-resistance, to identify the novel targets, to develop rational therapeutic strategies for overcoming flu-resistance and to provide new therapeutic options.

Drug-resistance is still one of the most pressing problems in treating cancer. Overexpression of some proteins [such as P-glycoprotein (P-gp), also known as ATP-binding cassette sub-family B member, multidrug resistance protein or cluster of differentiation 243 (CD243)] or alteration of some genes (such as p53) leads to the aberrant cell signaling and dysregulation of cell function [15, 16]. Sphingolipids are a class of lipids with important functions involved in a variety of cellular processes such as growth, proliferation, differentiation, senescence, apoptosis, survival and drug-resistance [17–20]. The metabolism of sphingolipids is one of the important signaling pathways that regulate apoptotic (chemotherapy), survival (drug resistance) and proliferative (cancer progression) activities [17–20]. Deregulation of sphingolipid metabolism is reflected in various pathophysiological conditions including metabolic disorders and cancers [17–20]. Ceramide, the central molecule of sphingolipid metabolism, generally mediates anti-proliferative and pro-apoptotic functions, and has important therapeutic potential [21]. A number of anticancer drugs or cytotoxic agents can significantly induce the accumulation of ceramide in response to treatment [19]. On the contrary, ceramide can also be converted to glucosylceramide by glucosylceramide synthase (GCS) which transfers the glucose from uridine diphosphate glucose to ceramide, promptly decreasing ceramide levels and consequently promoting cell survival [18, 19]. It is very important to understand how ceramide metabolism is associated with drug-resistance.

In the present study, we isolated the peripheral blood mononuclear cells (PBMCs) from 34 CLL patients, treated them with flu, and analyzed cell viability to identify primary flu-refractory and flu-relapsed patients. We used MEC-2 cells, a CLL cell line established from the peripheral blood of a patient with B-chronic lymphocytic leukemia [22], to establish flu-resistant clonal cells and demonstrated that flu-resistance is associated with the alteration of ceramide metabolism and the development of leukemia stem cell (LSC)-like cells, and that the modulation of ceramide metabolism can enhance flu sensitivity and reverse flu resistance.

## RESULTS

### Effect of flu on PBMC viability

We isolated the PBMCs from 34 CLL patients: 14 patients are chemo-naïve and 20 patients were treated with either single drug (alemtuzumab, rituximab, ofatumumab, pembrolizumab, bendamustine, ibrutinib or idelalisib) or combinations (bendamustine and rituximab; flu and rituximab; cyclphosphamide, vincristine and prednisone or FCR). The isolated PBMCs were treated with 10 µM flu for 72 hrs and then measured cell viability. Table 1 showed patient prognostic, pretreatment characteristics and cell viability. We found four flu-insensitive patients which cell viability is over 85%. Two are chemo-naïve patients (P7 and P21), one is bendamustine-rituximab-treating patient (P3) and the other is FCR-treating patient (P19). Due to the limited amount of patient blood samples and most of the PBMCs only survive but do not proliferate *in vitro*, we used MEC-2 cells, a CLL cell line, to establish flu-resistant clonal cells and to study molecular mechanism of flu-resistance.

**Table 1:** Patient prognostic, pretreatment characteristics and cell viability

Patient	Sex/Age	IgHV	ZAP70	CD38	Genetic alterations	Pretreatment	Cell viability (% of control)
1	M/41	M			del17p	Pemb	45.9
2	M/70	UM	5%	2%	del11q, del13q	BR	33.4
3	M/51	UM			trisomy12	BR	86.8
4	M/70				del13q	Ibru	21.4
5	F/81		5%		del13q, Trisomy12	R-CVP, Ibru	54.2
6	M/67		42%	42%	trisomy12	On observation	48.7
7	M/57	n.d.			n.d.	On observation	90.9
8	M/68	M	100%	neg	Y(6q23 del)	BR	22.4
9	M/88	n.d.			del13q	R	13.2
10	F/79	n.d.			del13q	BR, Ibru	33.0
11	M/78	n.d.			trisomy12	On observation	20.0
12	M/57	n.d.	n.d.	n.d.	del13q	On observation	28.8
13	M/85	M	0%	72%	n.d.	On observation	15.1
14	M/66	n.d.	n.d.	n.d.	normal	CVP, BR, Ibru	38.6
15	M/60	n.d.	18%	60%	del13q, del17p	FCR, Ibru	16.9
16	F/85	M	n.d.	n.d.	trisomy12	On observation	28.9
17	F/91	n.d.	n.d.	n.d.	del17p, Trisomy12	BR, R	30.5
18	F/60	n.d.	n.d.	14%	del13q	FCR, R, Ibru	34.9
19	M/48	UM	100%	2%	del13q	FCR	149.8
20	M/63	M	n.d.	9%	del13q	On observation	21.6
21	M/53	n.d.	100%	3%	normal	On observation	87.8
22	F/65	UM	87%	3%	normal	On observation	33.8
23	M/65	n.d.	n.d.	n.d.	n.d.	Alem, Ibru	57.6
24	F/63	M	0%	8%	del13q	On observation	30.3
25	M/67	n.d.	50%	9%	normal	On observation	29.7
26	M/62	n.d.	36%	0%	del13q	BO, Ibru, Idela	63.1
27	M/72	n.d.	n.d.	22%	del13q, del17p	Ibru	41.8
28	M/50	n.d.	38%	18%	del17p	Ibru	34.7
29	M/71	M	n.d.	n.d.	del13q	Radiation	17.6
30	M/40	UM	pos		normal	FCR	50.7
31	M/64	n.d.	n.d.	n.d.	normal	On observation	34.9
32	F/72	n.d.	n.d.	neg	del13q	FR	8.6
33	M/82	n.d.	n.d.	n.d.	del13q	On observation	47.8
34	M/67	UM	n.d.	neg	del13q	On observation	56.9

Notes: F, female; M, male or mutated; UM, unmutated; n.d., not determined; neg, negative; pos, positive; Pemb, Pembrolizumab; B, bendamustine, R, rituximab; Ibru, ibrutinib; Alem, alemtuzumab; Ide, idelalisib; O, ofatumumab; CVP, cyclophosphamide, vincristine and prednisone; and FCR, fludarabine, cyclophosphamide, and rituximab.

### Establishment and characteristics of flu-resistant clones

**Figure 1:**
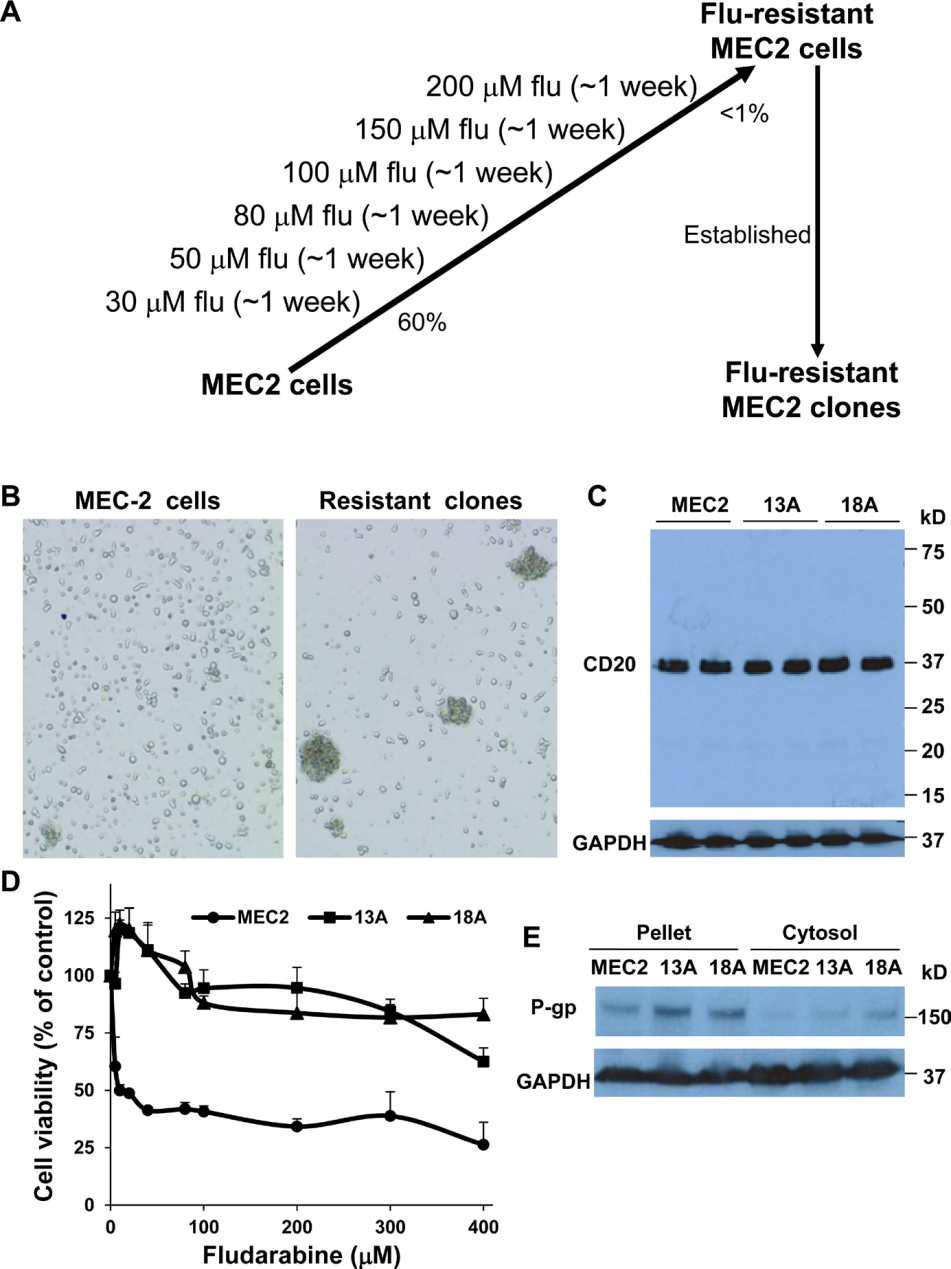
Characterization of flu-resistant clones (**A**) Establishment of flu-resistant clonal cells. MEC-2 cells were cultured in the media containing escalating flu concentrations (from 30 to 200 µM). The cells were maintained in 200 µM flu-containing medium for 1 week. Up to > 99% cell death, the rest cells were cloned by 96-well plates and grown for weeks. The flu-resistant clonal cells were maintained in the medium containing 100 µM flu. (**B**) Phase contrast microscopy of MEC-2 cells and flu-resistant clonal cells (clones 13A and 18A). (**C**) Expression of CD20. MEC2 cells and flu-resistant clonal cells were harvested and lyzed, and equal amount of cellular proteins was processed for immunoblotting using the antibodies against CD20 and GAPDH. (**D**) Determine LD_50_ of flu in MEC2 cells and flu-resistant clonal cells. The cells were treated with different concentrations of flu for 72 hrs and cell viability was determined by MTT (*n* = 16). (**E**) Expression of P-gp. Equal amount of cellular proteins from pellet or cytosol from MEC2 cells and flu-resistant clonal cells was processed for immunoblotting using the antibodies against P-gp and GAPDH. The data for B, C and E represent duplicate samples in at least three experiments.

Using escalating concentrations of flu (from 30 µM up to 200 µM), we have established multiple MEC-2 flu-resistant clones (Figure 1A). Flu-resistant clonal cells did not display any obvious morphological changes, except they grew in large clumps (Figure 1B). To determine the B cell lineage of flu-resistant clonal cells, we performed immunoblotting to compare expression of CD20, a B-cell marker, in MEC-2 cells and flu-resistant clonal cells (clones 13A and 18A). Figure 1C illustrated that expression of CD20 levels in MEC-2 cells and flu-resistant clonal cells was similar. Next, we determined the effect of flu concentrations on cell viability of MEC-2 cells and flu-resistant clonal cells. Figure 1D shows that the clonal cells are clearly resistant to flu-treatment. The lethal dose 50 (LD_50_) in MEC-2 cells is 13.5 ± 2.1 µM, but >400 µM in flu-resistant clonal cells. To confirm flu-resistance, we analyzed expression of P-gp which is a drug-resistant marker and can pump drug out of cells [15]. Expression of P-gp was significantly up-regulated in flu-resistant clonal cells (Figure 1E). These two lines of evidence demonstrate that our selected clonal cells are flu-resistant cells.

### Flu-treatment induces apoptosis in MEC-2 cells but not in flu-resistant clonal cells

**Figure 2:**
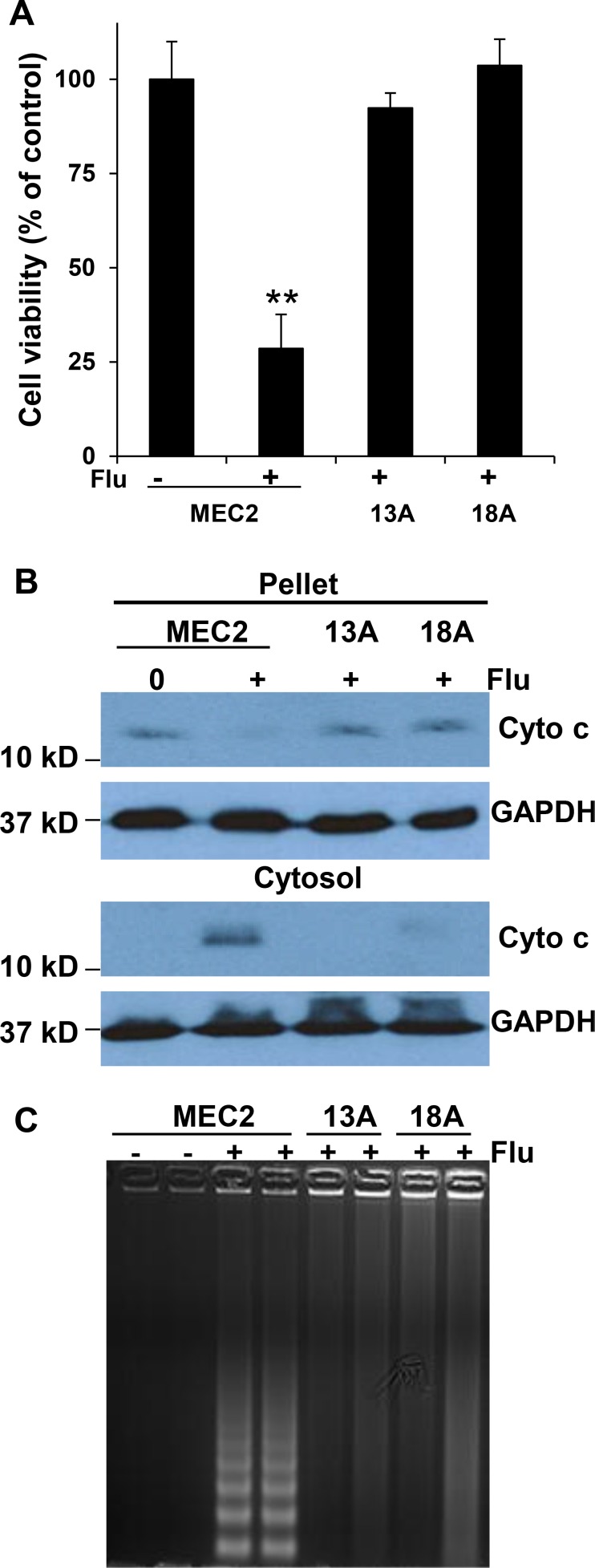
Flu induces MEC-2 cell apoptosis but not flu-resistant clonal cells (**A**) Cells were treated with or without 100 µM flu for 72 hrs and cell viability was analyzed by MTT (*n* = 16). The value of treatment was statistically different from the controls. ^**^P < 0.01. (**B**) Cells were fractionated to yield the pellet and cytosol, and equal amounts of cellular protein from the pellet and cytosol were processed for immunoblotting using the antibodies against cytochrome c (Cyto c) and GAPDH. (**C**) The cells were treated with or without 100 µM flu concentrations for 24 hrs. The cells were collected and lysed to prepare total DNA, and the samples were separated on a 1.2% agarose gel. The data for B and C represent triplicate samples in three experiments.

**Figure 3:**
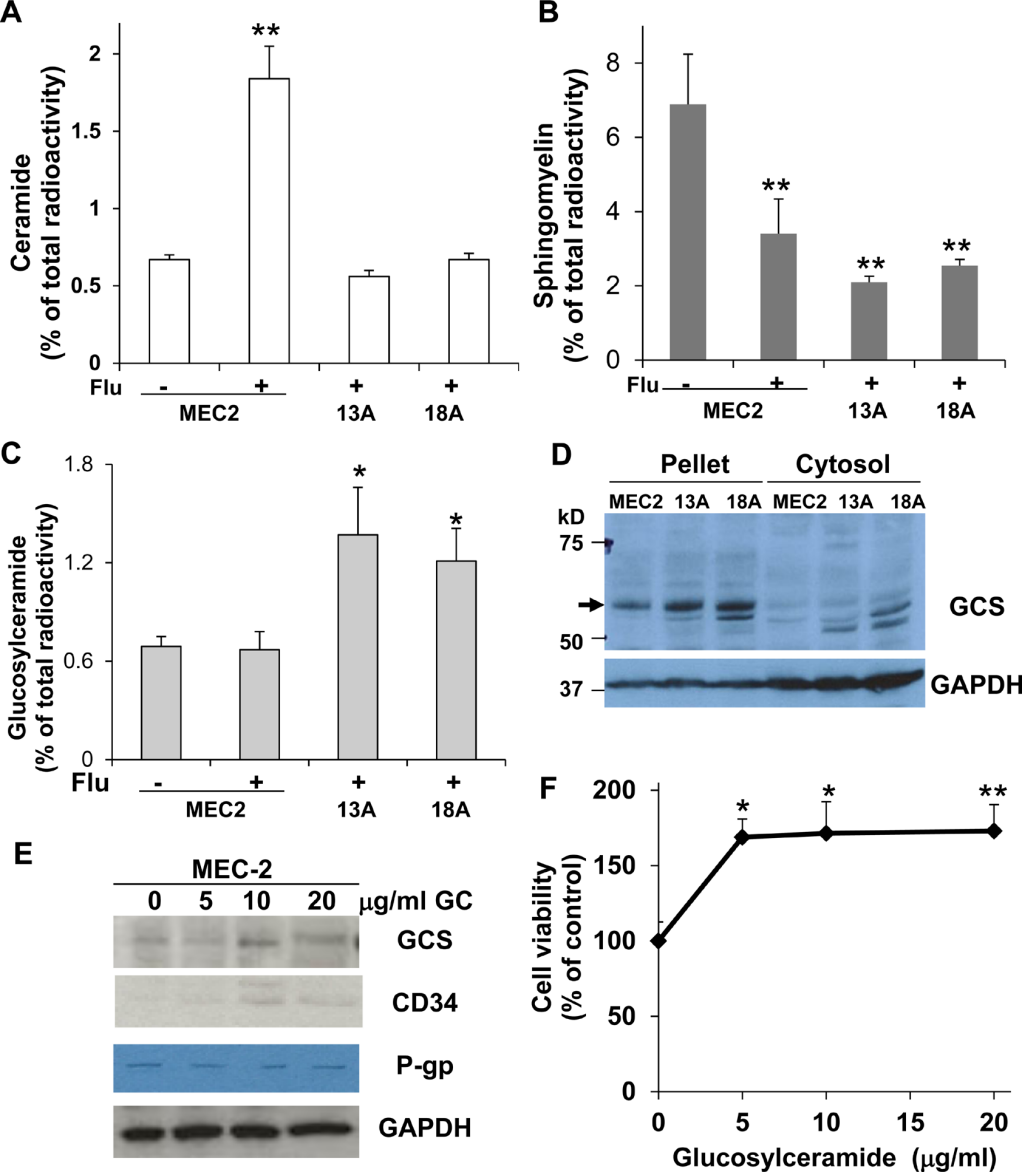
The formation of ceramide and glucosylceramide and the expression of GCS in MEC-2 cells and flu-resistant clonal cells The cells were prelabeled with [^3^H]palmitic acid for 24 hrs and then treated with or without 100 µM flu concentrations for 24 hrs. Total cellular lipids were extracted and analyzed for the accumulation of [^3^H]ceramide (**A**), the degradation of [^3^H]sphingomyelin (**B**), and the formation of [^3^H]glucosylceramide (**C**). (**D**) The cells were harvested and processed for immunoblotting using antibodies against GCS and GAPDH. MEC-2 cells were treated with different concentrations of glucosylceramide for 24 hrs, and the cells were analyzed for GCS, CD34, P-gp and GAPDH expression (**E**) and cell viability (**F**). The data represent triplicate samples in three experiments. The values of treatment were statistically different from the controls. ^*^P < 0.05. ^**^P < 0.01.

Earlier studies showed the involvement of caspase activation and ceramide accumulation in flu-induced apoptosis of B-cell leukemia cell lines (WSU and JVM-2 cells) and Jurkat lymphoblastic leukemia cells [23, 24]. In order to investigate whether flu-resistance is associated with ceramide metabolism, we firstly determined whether flu induces MEC-2 cell apoptosis and ceramide accumulation. Figure 2A showed that flu treatment significantly reduced parental MEC-2 cell viability but not flu-resistant clonal cells. Flu treatment induced apoptotic processing was analyzed by cytochrome c release and DNA cleavage. Figure 2B and 2C illustrated that flu treatment induced cytochrome c release and DNA cleavage in MEC-2 cells but not in flu-resistant clonal cells. We next determined whether flu-induced apoptosis is associated with ceramide accumulation. MEC-2 cells and flu-resistant clonal cells were prelabeled with [^3^H]palmitic acid and treated with or without flu. Figure 3A shows the accumulation of [^3^H]ceramide in flu-treated MEC-2 cells but not in control and flu-resistant clonal cells. The data based on ceramide accumulation, cytochrome c release, DNA cleavage and the reduction of cell viability indicate that flu-induced ceramide is associated with apoptosis in MEC-2 cells, but flu-induced apoptosis does not occur in the flu-resistant clonal cells.

### Accumulation of glucosylceramide and overexpression of glucosylceramide synthase in flu-resistant clonal cells

Ceramide, a product of sphingomyelin degradation, can induce cell programmed death [21] and can also be converted to other non-cytotoxic metabolites, such as glucosylceramide, which has the effect of promptly eliminating ceramide level and consequently promoting cell survival [17–19]. In examining [^3^H]sphingomyelin degradation, we found similar degradation of [^3^H]sphingomyelin in flu-treated MEC-2 cells and flu-resistant clonal cells (Figure 3B) although the accumulation of [^3^H]ceramide was not observed in flu-resistant clonal cells (Figure 3A). The results indicate that the formation of ceramide in flu-resistant clonal cells is likely converted to other metabolites. To identify possible metabolites, we analyzed the same samples and found a significant increase of [^3^H]glucosylceramide in flu-resistant clonal cells (Figure 3C). To determine whether the accumulation of glucosylceramide is associated with GCS overexpression or activation, we further performed immunoblotting to determine expression of GCS in MEC-2 cells and flu-resistant clonal cells. Figure 3D clearly shows that GCS expression is up-regulated in flu-resistant clonal cells. Next, we treated MEC-2 cells with different concentrations of glucosylceramide for 24 hrs and then determined the effect of glucosylceramide on GCS expression and cell proliferation. The results showed that glucosylceramide enhanced expression of GCS and CD34 (Figure 3E) and promoted cell proliferation (Figure 3F).

### PDMP inhibits the formation of glucosylceramide and restores chemo-sensitivity in flu-resistant clonal cells

**Figure 4:**
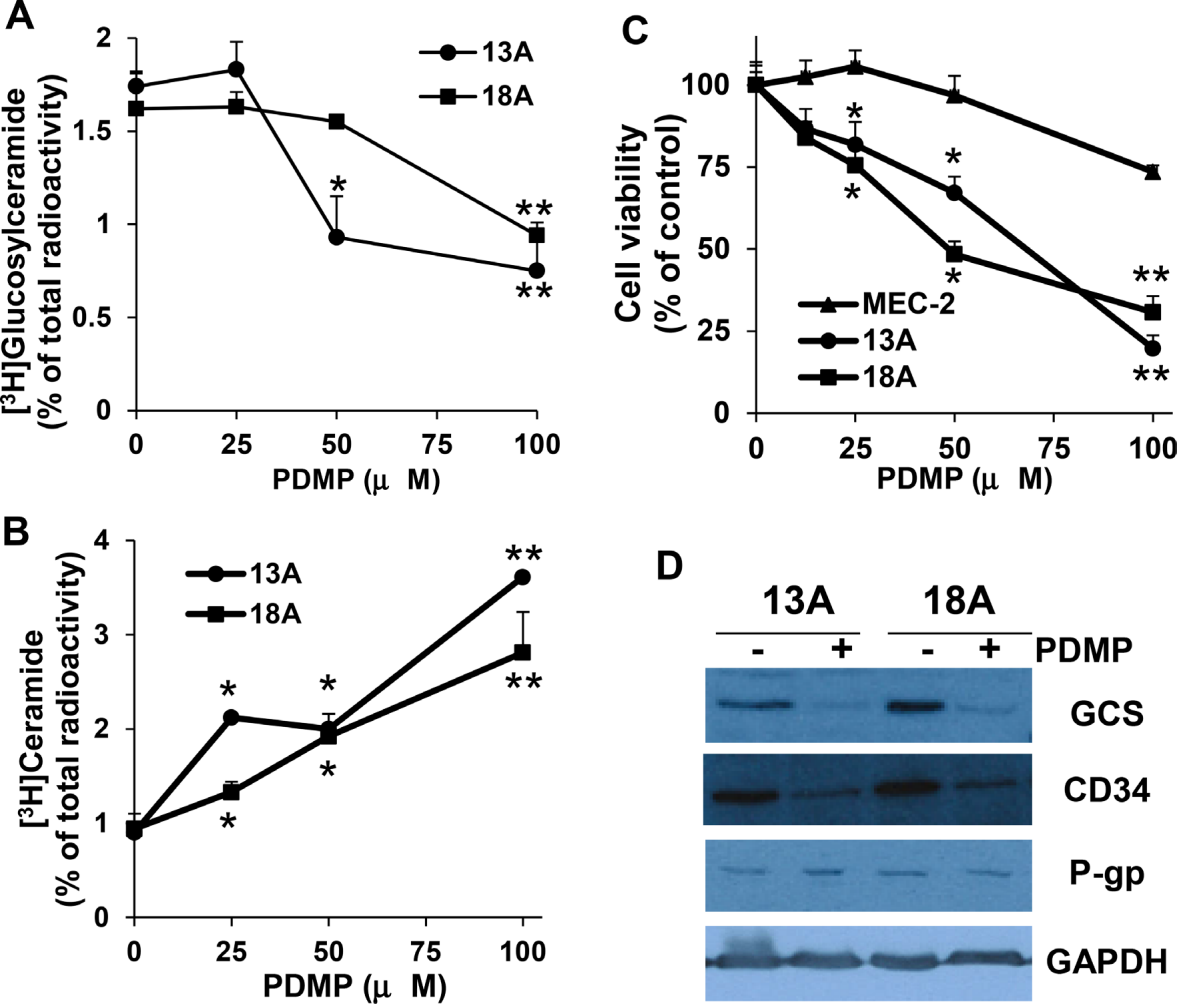
Effect of PDMP on the formation of ceramide and glucosylceramide, cell viability, and GCS expression Flu-resistant clonal cells (clones 13A and 18A) were prelabeled with [^3^H]palmitic acid for 24 hrs in 100 µM flu-containing medium and then treated with different concentrations of PDMP for 24 hrs. Total cellular lipids were extracted and [^3^H]glucosylceramide (**A**) and [^3^H]ceramide (**B**) were analyzed. (**C**) Cells were treated with different concentrations of PDMP for 72 hrs and cell viability was analyzed by MTT (*n* = 16). (**D**) Flu-resistant clonal cells were treated 50 µM PDMP for 24 hrs, the cells were harvested and processed for immunoblotting against GCS, CD34, P-gp and GAPDH. The flu-resistant clonal cells are in the medium containing 100 µM flu. The data for A and B represent triplicate samples in three experiments. The concentrations of PDMP that caused a significant alteration in the accumulation of ceramide metabolite compared with the controls were statistically presented. ^*^P < 0.05. ^**^P < 0.01.

Our results indicate that the conversion of ceramide to glucosylceramide is clearly increased in flu-resistant clonal cells. To further confirm whether this conversion is associated with CLL cell flu-resistance, we use PDMP to block the conversion of ceramide to glucosylceramide. PDMP is a ceramide analog and can block the glycosylation of ceramide by inhibiting GCS [25]. The cells were prelabeled with [^3^H]palmitic acid for 24 hrs, and then incubated with different concentration of PDMP in 100 µM flu-containing medium for 24 hrs. Total cellular lipids were extracted and analyzed for the formation of [^3^H]glucosylceramide and [^3^H]ceramide. Figure 4A and 4B showed that PDMP clearly inhibited the activity of GCS leading to the reduction of [^3^H]glucosylceramide and the accumulation of [^3^H]ceramide in flu-resistant clonal cells. To more directly assess the association of glucosylceramide with flu-resistance, we further analyzed the effect of PDMP on cell viability of flu-resistant clonal cells. As shown in Figure 4C, treatment of flu-resistant clonal cells with PDMP significantly reduced cell viability. To further confirm the association of glucosylceramide with flu-resistance, flu-resistant clonal cells were treated with 50 µM PDMP for 24 hrs and the cells were processed for immunoblotting to determine GCS expression. Figure 4D clearly showed that PDMP not only inhibited the formation of glucosylceramide but also reduced expression of GCS and CD34. Taken together, our data support that the accumulation of glucosylceramide is associated with flu-resistance and reducing cellular glucosylceramide can restore flu-sensitivity.

### Flu-resistant clonal cells are LSC-like cells

**Figure 5:**
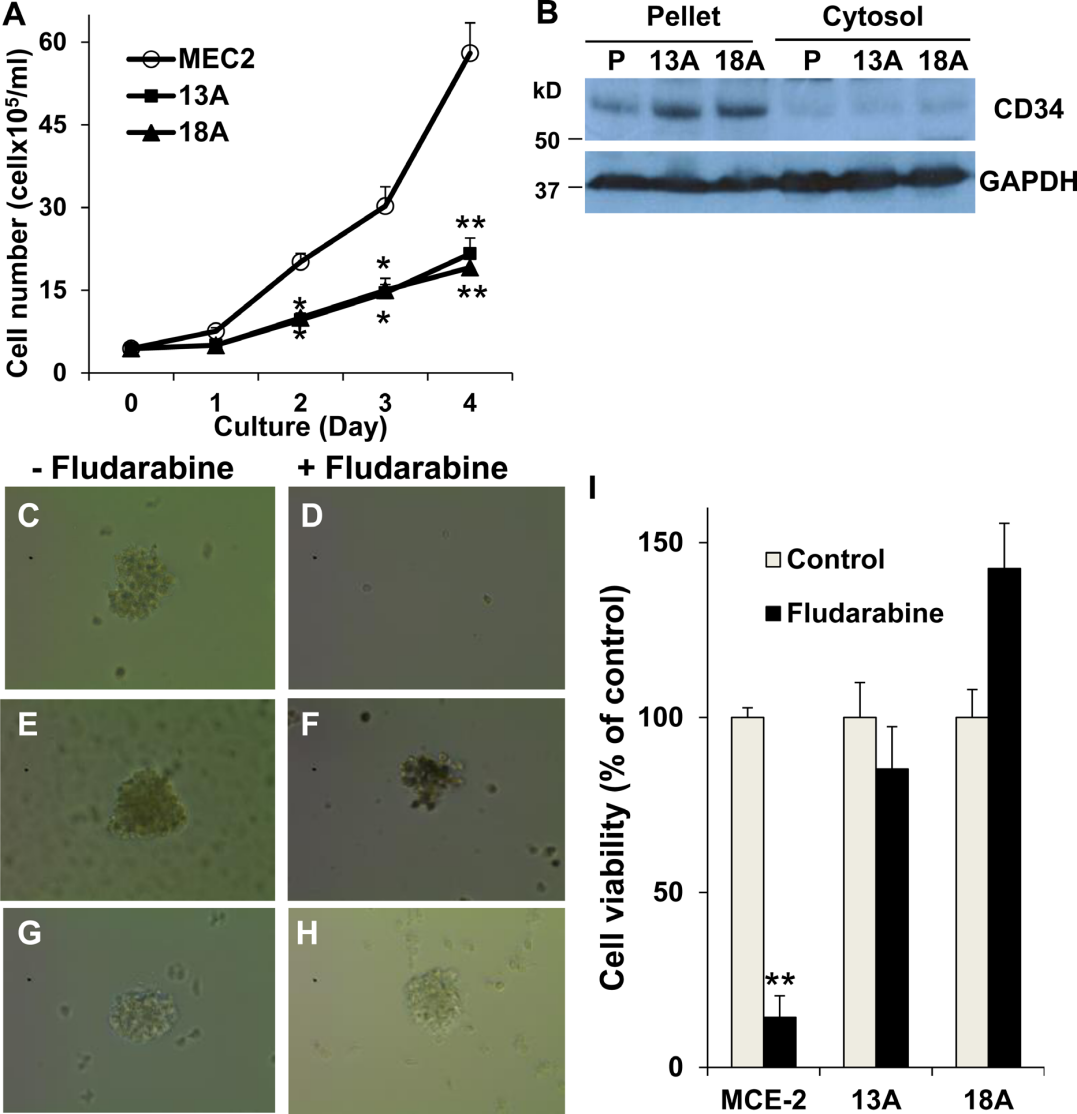
Characterization of LSC property in flu-resistant clones (**A**) Cell growth curve. Equal numbers (1 x 10^5^ cells/well) of MEC-2 cells or flu-resistant clonal cells were cultured in 12-well plates and counted every day for 4 days, and the resultant cell numbers generated a cell growth curve and calculated population doubling time (*n* = 8). (**B**) Expression of CD34. MEC-2 cells and flu-resistant clonal cells were harvested and fractionated. Cellular fractions were processed for immunoblotting using the antibodies against CD34 and GAPDH. (**C**–**H**) Images of forming colonies from MEC-2 cells (C and D) and flu-resistant clonal cells (13A, E and F; 18A, G and H). Cells were cultured in Methocult medium with or without flu for 14 days and the colonies were photographed by AMG EVOS Core Cell Imaging System. (**I**) Quantification of colonies. The colonies in the wells were determined by MTT (*n* = 14). The data represent multiple samples in two or three experiments. The values of cell number were statistically different from the original number. ^*^P < 0.05. ^**^P < 0.01.

Cancer stem cells are a small subpopulation of cancer-initiating cells that tend to be drug resistance and have the capabilities of self-renewal, proliferation, differentiation and tumorigenicity [26, 27]. To test whether flu-resistant clonal cells are LSC-like cells, MEC-2 cells and flu-resistant clonal cells were seeded at a density of 1 × 10^5^ cells into 12-well plates, and counted at 1, 2, 3 and 4 days after seeding. The resultant cell numbers generated a cell growth curve and calculated population doubling time. By comparison MEC-2 cells to flu-resistant clonal cells, we found slower growth in flu-resistant clonal cells (Figure 5A). The flu-resistant clonal cells exhibited doubling times of ∼30 ± 1.4 hours that were significantly longer than the 22 ± 2.1 hour doubling time measured for MEC-2 cells. It is clear that flu-resistant clonal cells have slow-growing and self-renewal capacity. We further analyzed expression of CD34, a marker antigen expressed on the surface of LSCs [26, 27], in MEC-2 cells and flu-resistant clonal cells. Figure 5B shows that expression of CD34 is significantly up-regulated in flu-resistant clonal cells (Figure 5B). To confirm that flu-resistant clonal cells are LSC-like cells, we used methylcellulose-based medium for colony formation. Figure 5C–5H showed the colonies of MEC-2 cells and flu-resistant clonal cells in the presence or absence of flu. More colonies were found in flu-resistant clonal cells compared to parental MEC-2 cells, in particular with flu treatment (Figure 5I). Based on slow-growing, self-renewal capacity, up-regulation of CD34 and colony formation, flu-resistant clonal cells compared to parental cells tend to be more LSC-like cells.

### Overexpression of GCS and CD34 in flu-insensitive PBMCs

**Figure 6:**
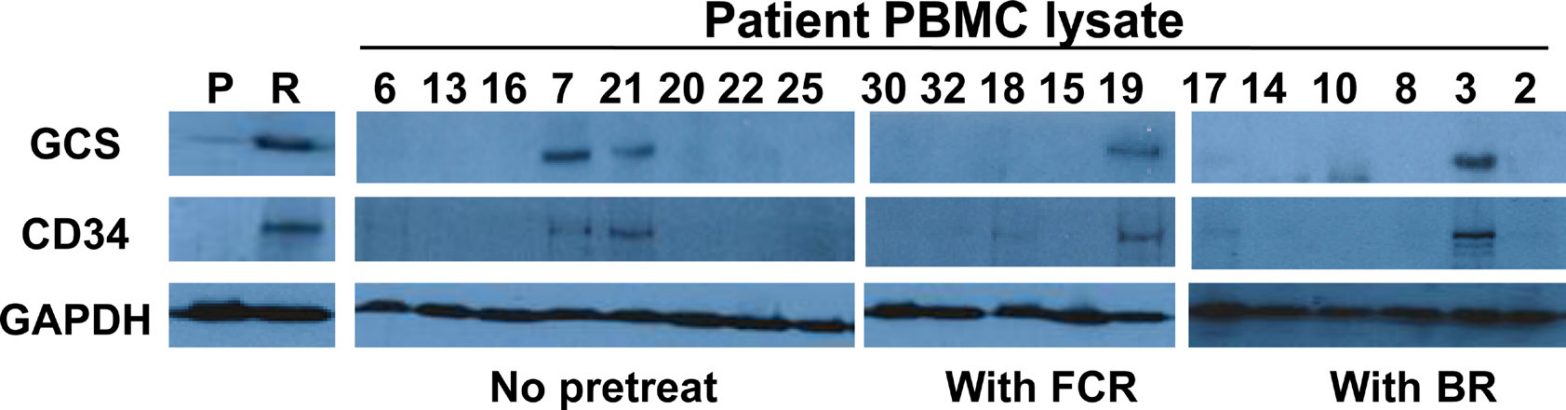
Expression of GCS and CD34 in the PBMCs from different CLL patients Equal amount of cellular proteins from MEC-2 cell (P), flu-resistant cell (R) and patient PBMC lysates were processed for immunoblotting using the antibodies against GCS, CD34 and GAPDH. The patients are no treated, BR treated or FCR treated. Lane numbers are patient numbers showed in Table 1.

As shown in Table 1, the PBMCs from four CLL patients are flu-insensitivity. We lyzed multiple CLL patient’s PBMCs from chemo naïve, treated with either the combination of bendamustine and rituximab or FCR, and then the samples were processed for immunoblotting to determine the expression of GCS and CD34. We found that expression of GCS or CD34 was significantly up-regulated in flu-insensitive samples compared to flu-sensitive samples (Figure 6). These results are similar to flu-resistant clonal cells, and indicate that flu-insensitivity in PBMCs is also associated with the alteration of ceramide metabolism and the development of LSC-like cells.

## DISCUSSION

The resistance to flu-based therapies is one of the predominant reasons for treatment failure and is a major challenge for CLL treatment. In analyzing CLL patients resistant to flu, Moussay *et al.* found various genomic abnormalities (deletion or gain) in more than twenty genes that are involved in p53, DNA damage and repair, cell cycle and apoptosis signaling [28]. Using piggyBac transposon-mediated mutagenesis combined with next-generation sequencing, one recent report also found that some new candidate genes such as deoxycytidine kinase and BMP-2-inducible protein kinase could be associated with flu-resistance in HG3 cells, a human modified CLL cell line [29]. Identifying the genes that are involved in flu-resistance is important because these cytogenetic mutations may be prognostic markers. However, genomic alterations are not enough because the proteins coded by these genes are involved in multiple different signaling pathways that can play opposite roles in the regulation of cellular functions. Understanding the signaling pathways is a key to develop new strategies for overcoming flu-resistance.

There are few CLL cell lines available for research. Current several flu-resistant cell lines, such as malignant B-1 cell line (a mouse model of CLL) [30], K562 cells (a cell lines from chronic myelogenous leukemia patient) [31] and HG3 cells (a human modified CLL cell line) [29], are not ideal cell model for defining flu-resistant signaling pathways because these cell lines are either a modified cell line or not human CLL cell lines. Here, we used MEC-2 cells, a cell line established from the peripheral blood of a patient with B-chronic lymphocytic leukemia [22] to establish flu-resistant CLL clonal cells and used the clonal cells as a platform to study molecular mechanism of flu-resistance. Our recent study shows that MEC-2 cells respond to flu treatment similarly to the PBMCs from CLL patients [32]. By comparing parental cells to flu-resistant clonal cells, we found that flu-resistant clonal cells like their parent cells express very high CD 20, a B-cell CD marker, but the flu-resistant clonal cells exist the significant alteration of ceramide metabolism that is associated with overexpression of GCS and the development of LSC-like cells that up-regulates CD34 expression. Importantly, up-regulation of GCS and CD34 expression was also found in flu-resistant PBMCs from CLL patients (Figure 6). This could be proved by the fact that the conversion of ceramide to glucosylceramide in CLL cells plays a key role in flu-resistance.

Ceramide induced by numerous apoptotic stimuli (e.g. cytokines, anticancer drugs or cytotoxic agents, irradiation and environmental stresses) is recognized as a proapoptotic signaling molecule. Increasing the levels of cellular ceramide can enhance many proapoptotic molecules such as NH_2_-terminal Jun kinase, caspase-3, and reactive oxygen species [20] and suppress antiapoptotic molecules such as phosphatidylinositol 3-kinase, AKT and mTOR [33, 34]. Comparing to parental cells, our data clearly showed that flu-resistant clonal cells altered ceramide metabolism and up-regulated GCS expression (Figure 3 and Figure 6). Schwamb *et al.*. identified BCR engagement to catalyze the crucial modification of ceramide to glucosylceramide in drug-resistance of primary CLL cells [35]. Earlier reports indicate that glucosylceramide can stimulate DNA synthesis and cell growth (Figure 3F) [36, 37]. More and more evidence supports the accumulation of glucosylceramide in multidrug resistant cancer cell lines isolated from different solid tumors [38, 39]. Overexpression of GCS was also reported in adriamycin-resistant K562 cells, vincristine-resistant HL-60 cells and clinical multidrug resistant samples of acute myeloid leukemia, acute lymphocytic leukemia and chronic myeloid leukemia [40–43]. Our results and the data from many others [38–43] indicate the biochemical significance of accumulation of glucosylceramide and overexpression of GCS in drug-resistant cancer cells, and the inhibition of GCS has therapeutic potential for restoration of chemo-sensitivity and reversal of drug-resistance.

Our data demonstrate that overexpression of GCS alters ceramide metabolism and promotes cancer cell survival. P-gp is the first described and most extensively studied multidrug resistant efflux protein that results in resistance to many structurally unrelated drugs [44]. Earlier studies showed that glucosylceramide is a substrate for P-gp and that both ceramide and glucosylceramide regulate P-gp expression and function [41, 45–47]. Using a dithionite fluorescence quenching technique, Eckford *et al.* [45] showed that P-gp is a broad-specificity outwardly-directed flippase which enhances glycosphingolipid translocation. On the other hand, both cyclosporin A and GF120918 (p-gp inhibitors) can increase C8-ceramide mediated apoptosis [46]. Using siRNA to silence GCS, knockdown of GCS expression affects P-gp expression and function [42]. All these data clearly shows that either ceramide or glucosylceramide plays an important role in the regulation of P-gp expression and function [47]. We found up-regulation of P-gp expression in flu-resistant clonal cells (Figure 1E), but the modulation of glucosylceramide levels by adding or depleting glucosylceramide does not significantly regulate P-gp expression in MEC-2 cells and flu-resistant clonal cells (Figures 3E and 4D). Whether P-gp expression is regulated by ceramide metabolites and whether P-gp interacts with GCS need to be further studied.

Cancer stem cells were first identified in myeloid leukemia with the cell surface marker combination of CD34^+^ and CD38- [48]. These cells exhibit a slowing growth and pronounced self-renewal and differentiation capacity. Recently, accumulating evidence supports that cancer stem cells are considered as a major source of cancer recurrence and therapeutic resistance [49–51]. Based on overexpression of CD34, slow-growth and self-renewal capacity and colony formation (Figure 5), we conclude that flu-resistant clonal cells are LSC-like cells. One recent study shows that glucosylceramide synthase is enhanced in breast cancer stem cells but not in normal mammary epithelial stem cells [52]. With the accumulation of glucosylceramide and up-regulation of GCS and CD34 expression in flu-resistant clonal cells, it indicates that ceramide metabolism is likely associated with the development of LSC.

**Figure 7:**
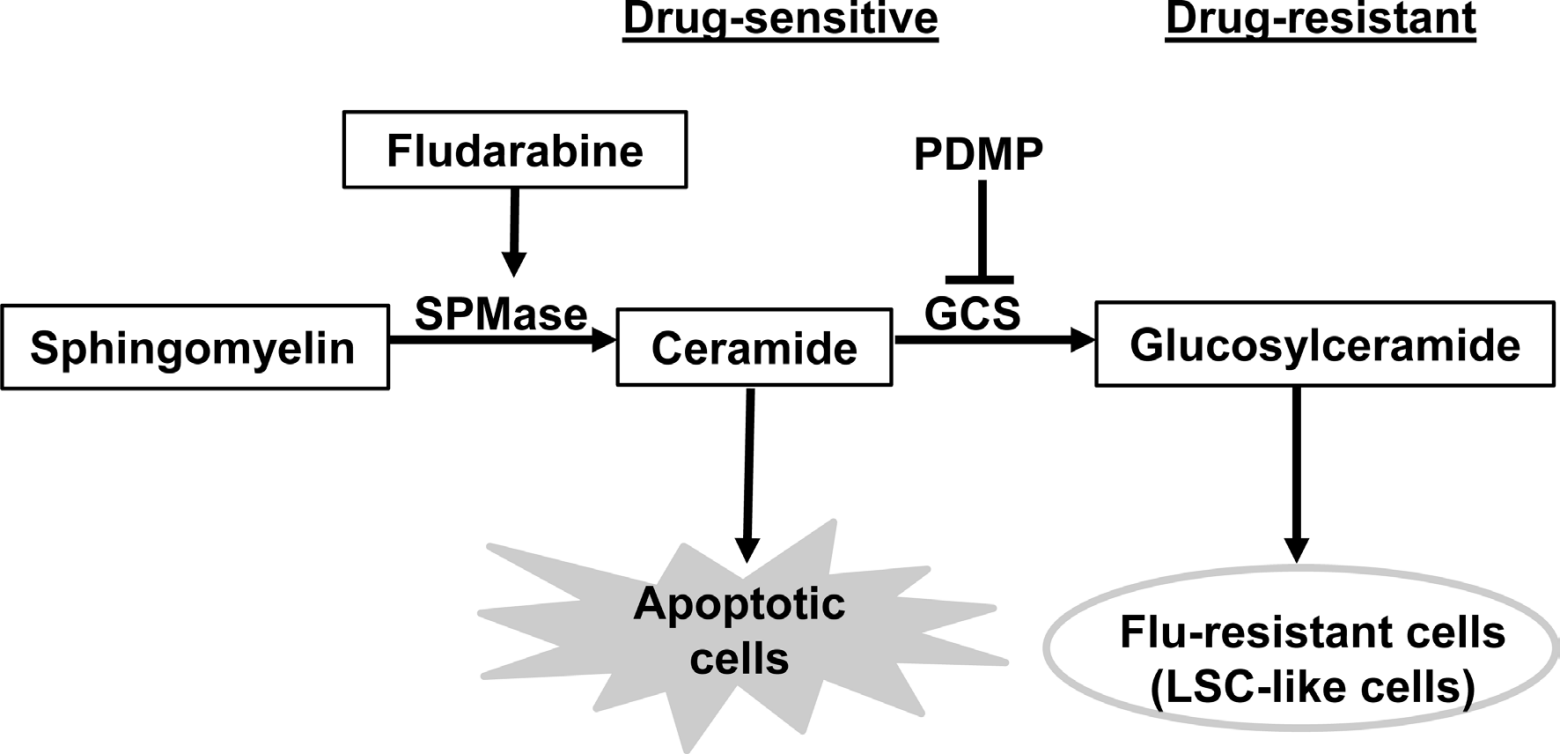
Signaling pathways that are involved in flu-resistant CLL cells Ceramide metabolism plays a key role in CLL cell apoptosis or flu-resistance. The increasing ceramide in flu-treated CLL cells leads to apoptosis but the conversion of ceramide to glucosylceramide in flu-resistant clonal cells can promote cell survival under flu-treatment. The ceramide metabolism is also associated with the development of LSC-like cells. SPMase, sphingomyelinase; GCS, glucosylceramide synthase.

In conclusion, our data present signaling pathways that are involved in flu-resistance (Figure 7) and show consistent evidence that flu-resistant clonal cells are associated with ceramide metabolism (decreasing ceramide level and increasing glucosylceramide level) and that reducing GCS expression and activity can reverse flu-resistance and restore drug-sensitivity. Moreover, flu-resistance is also associated with the up-regulation of CD34 expression which links to the development of LSC-like cells.

## MATERIALS AND METHODS

### Materials

All chemicals were purchased from Fisher Scientific (Pittsburgh, PA) or Sigma Chemicals (St. Louis, MO) unless specified otherwise. Cell culture reagents were provided by HyClone. (Logan, UT). Methylcellulose-base medium (MethoCult H4434 classic) was purchased from Stem Cell Technologies (Cambridge, MA). Flu and PDMP were obtained from Cayman Chemical (Ann Arbor, MI). Glucosylceramide was supplied by Matreya, LLC (College Station, PA). Ceramide was purchased from Avanti Polar Lipids, Inc. (Alabaster, Alabama). [^3^H]palmitic acid (30–60 Ci/mmol) were obtained from American Radiolabeled Chemicals, Inc. (St Louis, MO). Ficoll-Paque Plus was obtained from Amersham Biosciences (Piscataway, NJ, USA). The monoclonal anti-CD20 (L26), anti-GAPDH (0411), and anti-cytochrome c (7H8) antibodies, and the polyclonal anti-UGCG (glucosylceramide synthase, H-300) and anti-CD34 (H-140) antibodies were provided by Santa Cruz Biotechnology, Inc. (Dallas, TX). The monoclonal anti-P-glycoprotein (F4) antibody was supplied by Sigma-Aldrich (Saint Louis, Mo). CellTiter 96^®^ Non-Radioactive Cell Proliferation Assay kit (MTT) was purchased from Promega (Madison, WI, USA). Halt protease and phosphatase single-use inhibitor cocktail, SuperSignal West Pico chemiluminescent substrate and BCA protein assay reagent were obtained from Thermo Scientific (Rockford, IL).

### PBMC isolation and treatment

Blood was obtained from CLL patients as defined by NCI96 criteria 28 [53] following a receipt of written informed consent under an IRB protocol approved by Saint Louis University. PBMCs were isolated from whole blood immediately following donation using Ficoll density gradient centrifugation. Isolated cells were plated in 96-well assay plates at a concentration of 10–50,000 cells (depend on patient cell numbers) per well in 100 µl of RPMI 1640 media with 10% FBS with or without 10 µM flu. The cells were cultured for 72 hrs, and then cell viability was determined using Promega’s CellTiter 96^®^ Non-Radioactive Cell Proliferation Assay kit (MTT) according to the manufacturer’s instructions [32]. Absorbance at 570 nm was recorded using a BioTek Epoch Reader (Winooski, VT). The rest of PBMCs from CLL patients were harvested and lysed for immunoblotting.

### Cell culture and establishment of flu-resistant clones

MEC-2 cells were cultured in RPMI 1640 media with 10% FBS. For establishing MEC-2 flu-resistant clones, different concentrations of flu were added to culture medium beginning at 30 µM flu, and increased to 50, 80, 100, 150 and 200 µM flu stepwise weekly. Up to 10 × LD_50_ selection (about 2 month), the flu-resistant cells survived at high concentration of flu. The cells were cloned in 96-well plates (100 cells in 10 ml medium and 0.1 ml per well) and established the flu-resistant clones. Flu-resistant clonal cells were maintained in RPMI 1640 medium with 10% FBS and 100 µM flu. Cell morphology was photographed by AMG EVOS Core Cell Imaging System.

### Cell treatment, immunoblotting and cell viability assay

MEC-2 cells were treated with or without 100 µM flu for 3 hrs and flu-resistant clonal cells were maintained in the regular medium containing 100 µM flu. After treatment, cells were harvested and washed once with 1 × PBS. The cells were homogenized in a buffer containing 20 mM Hepes, 2 mM MgCl_2_, 1 mM EDTA and 1 mM DTT with protease and phosphatase inhibitor cocktails and centrifuged at 14,500 rpm to yield a pellet and supernatant. Cell lysates and cellular fractions were measured for protein concentration using the BCA protein assay reagent with BSA as a standard, and then adjusted to equal amounts of cellular protein in 1 × loading buffer. The samples were boiled for 10 min and 15 µg/lane were subjected to SDS-PAGE, and processed for immunoblotting with the appropriate antibodies [54].

In the experiments for analyzing cell viability, MEC-2 cells and flu-resistant clonal cells were plated in 96-well assay plates at a concentration of 50,000 cells per well in 100 µl of culture medium with different concentrations of flu. The cells were treated for 72 hrs, and then cell viability was determined. MEC-2 cells were incubated with different concentrations of glucosylceramide for 24 hrs, and the samples were further processed for immunoblotting analysis. For PDMP-treated experiments, MEC-2 cells and flu-resistant clonal cells (in “maintaining” medium containing 100 µM flu) were cultured in 96-well assay plates with different concentrations of PDMP for 72 hrs and then analyzed for cell viability. In some experiments, flu-resistant clonal cells were cultured in 6-well plates and treated with 50 µM PDMP for 24 hrs, and the samples were used for immunoblotting analysis.

### Cell radiolabeling and lipid metabolite analysis

MEC-2 cells and flu-resistant clonal cells were cultured in 6-well plates containing 0.5 ml medium with 2 μCi/ml of [^3^H]palmitic acid and 0.5 ml medium with or without 100 µM flu. After 24 hr treatment, the cells in the medium were collected and centrifuged at 1,500 rpm for 5 mins. Total cellular lipids in the cells were extracted by chloroform: methanol: water (5.5:5.5:5, v/v). In some experiments, the cells were prelabeled with [^3^H]palmitic acid for 24 hrs in 100 µM flu-containing medium and then treated with different concentrations of PDMP for another 24 hrs. The individual radiolabeled lipid was resolved from the total cellular lipids by thin layer chromatography and identified by co-migration with commercial standards in different solvent systems: I) chloroform: acetic acid (90:10, v/v) for ceramide, II) chloroform: methanol: ammonium hydroxide (40:10:10, v/v) for glucosylceramide, and III) chloroform: methanol: acetic acid: and water (50:25:8:4, v/v) for sphingomyelin. The standards were visualized with iodine vapor, and the areas corresponding to ceramide, glucosylceramide or sphingomyelin were scraped into scintillation vials and quantitated by liquid scintillation spectrometry.

### Measurement of DNA fragmentation

MEC-2 cells were treated with or without 100 µM flu for 24 hrs and flu-resistant clonal cells were cultured in “maintaining” medium containing 100 µM flu. After treatment, cells (dead and alive) were harvested by centrifugation at 1,500 rpm for 2 mins, and the pellets were re-suspended in 0.5 ml of lysis buffer containing 5 mM Tris-HCl, pH 8.0, 20 mM EDTA, and 0.5% Triton X-100 and placed on ice for >60 mins. The samples were then centrifuged at 14,500 rpm for 20 mins, and the supernatant containing DNA cleavage products with the same amount of cellular proteins was precipitated by isopropyl alcohol for 15 hrs. The samples were centrifuged at 14,500 rpm for 20 mins, and the pellets were re-suspended in Tris-EDTA buffer with proteinase K and RNase A for 2–3 hrs at 37° C. DNA fragments were separated on a 1.2% agarose gel, visualized with ethidium bromide, and photographed using the Bio-Rad Image System.

### Colony forming unit setting and analysis

MEC-2 Cells and flu-resistant clonal cells were harvested, counted and adjusted to 2 × 10^4^ cell/ml. To set up colony forming assay, we removed 5 ml MethoCult medium to a set of tubes and then added 0.5 ml cell suspense to each tube. The cells were mixed with MethoCult medium by vortex, dispensed into 48-well plates and cultured in the 37° C. The 25 μl regular medium or 100 µM flu-containing medium was carefully added to the designed wells in day 1 and day 8. The colonies were photographed by AMG EVOS Core Cell Imaging System. To quantify the colonies, the plates were determined using Promega’s CellTiter 96^®^ Non-Radioactive Cell Proliferation Assay kit (MTT).

### Data analysis

The data were analyzed for significance using one-way repeated measures of ANOVA followed by Tukey’s test for comparisons between the experimental groups shown in the figures.

## Abbreviations

CLL: Chronic lymphocytic leukemia
FCR: fludarabine, cyclophosphamide and rituximab
flu: fludarabine
GCS: glucosylceramide synthase
LSC: leukemia stem cell
PBMCs: peripheral blood mononuclear cells
P-gp: P-glycoprotein
